# Global assessment of forest quality for threatened terrestrial vertebrate species in need of conservation translocation programs

**DOI:** 10.1371/journal.pone.0249378

**Published:** 2021-04-14

**Authors:** Jessica L. Roberts, W. Justin Cooper, David Luther

**Affiliations:** 1 Environmental Science and Policy Department, George Mason University, Fairfax, Virginia, United States of America; 2 Biology Department, George Mason University, Fairfax, Virginia, United States of America; 3 Smithsonian Mason School of Conservation, George Mason University, Fairfax, Virginia, United States of America; Sichuan University, CHINA

## Abstract

Conservation actions such as habitat protection, restoration, and translocations are critical actions in preventing further extinctions of threatened species. We used the 152 threatened species on the International Union for the Conservation of Nature’s Red List with conservation translocations as a recommended conservation action to access the habitat quality of these species’ ranges. We determined where multi-species conservation translocation and forest restoration efforts can be concentrated. To determine the habitat quality of species’ ranges, we assessed forest cover, forest restoration potential, protected area status, and invasive species concerns. Forty-four percent (67 species) of species with translocations recommended have part of their range in a protected area, existing forest cover, and currently no invasive species risk. However, the majority (85 species) currently need habitat management (63 species), invasive species control (71 species), or protection (34 species). We also identified key differences between species recommended for reintroductions (115 species) and benign introductions (37 species), such as the percentage of a species’ range within a protected area, in which reintroductions (median = 7.4%) had more than benign introductions (median = 0.9%). Mauritius, central Africa, eastern Australia and Himalaya regions each have areas with range overlap of three or more species recommended for translocations and forest restoration potential. For those species with CT programs in place, mean forest cover was 32% and restoration potential was 16%, suggesting potential minimum habitat requirements for initial releases. Results provide a global perspective on reintroduction and translocation needs of threatened species with evidenced-based information on habitat quality, i.e. forest restoration potential, forest cover, protected areas, and invasive species control, to aid conservation translocation scientists and ultimately improve the success of such projects.

## Introduction

Habitat loss is and will continue to be one of the main drivers of species population declines and extinctions for the foreseeable future [1–7]. Over 85% of the species listed under the International Union for the Conservation of Nature’s (IUCN) Red List (hear after Red List) of Threatened Species are experiencing population declines due to habitat loss and degradation [8, 9]. Unless global priorities change, this percentage is unlikely to improve due to the exacerbating effects of climate change and recent reductions in the legal protections and extent of protected areas [10–12]. Conservation actions such as habitat management, protection and species reintroductions could help conserve and rebuild threatened species populations [13, 14]. In fact, habitat management and protection are generally needed before species reintroductions can occur [8].

Conservation translocation (CT), defined as the “deliberate movement of organisms from one location to be released into another for intended conservation benefits,” is becoming an increasingly popular method of species restoration [15, 16]. Threatened species reintroductions are a subset of CT focused on releasing animals to an area within their historical range from which they were recently extirpated, while benign introduction is a second subset of CT where animals are released to an area outside of their historical range but still in an area deemed suitable for the species’ persistence [16]. Reintroductions and benign introductions are both valuable tools for reintroduction biologists, yet each is appropriate under different circumstances. In either case, the threats that initially spurred the species’ decline need to be alleviated, or suitable alternatives need to be found, before CT can be implemented to increase the likelihood of establishing self-sustaining populations. Unfortunately, CT success rates remain low [13, 15, 17–21]. Thus, there is still a need for more evidence-based and holistic information for better modeling, planning, and creating *a priori* objectives to increase success rates [15].

The IUCN’s Conservation Translocation Specialist Group’s *Guidelines for Reintroduction and other Conservation Translocations* outlines the importance of assessing a reintroduction area for its current and future ability to support the proposed CT species [16], including whether historical and current habitats are suitable or in need of management [15]. Though CT success can be based on several factors, habitat quality is known to be a key factor that influences post-release survival and establishment, leading to self-sustaining populations [13, 18, 21]. Habitat quality and suitability can involve many factors, such as the correct vegetative species and structure [22], availability of food [23], connectivity and distance from anthropogenic threats [24], and lack of invasive species [25]. While releasing animals into historical locations of a species’ original range is often the first option for CT [13, 26], habitats can change due to environmental and anthropogenic influences [26]. Thus, determining whether the historic ranges are suitable for CT is a logical first step for species restoration programs.

Forest biomes host a disproportionate number of threatened species; for example, 5,547 of the 6,680 mammals, birds, and amphibians assessed as threatened (Vulnerable, Endangered, or Critically Endangered) or Near Threatened on the Red List occur in forests [9]. CT of extirpated forest species has the potential to improve the conservation status and populations of many of these threatened species; however, the forest ecosystem needs to be adequately healthy and suitable for each species to facilitate establishment and persistence. Here we assess whether current forest habitats are suitable for species with designated CT programs. We assess the forest cover, land protection status, forest restoration potential, and invasive species control needs of threatened and Near Threatened species with CT as a recommended conservation action. In addition, we identify critical areas for potential reintroductions, which are areas where multiple species would benefit from CT and forest restoration in the same location, thus having the largest potential impact for species conservation. We provide a global approach to identify the best areas for possible forest species CT and optimize synergistic opportunities between CT and habitat restoration, thus contributing to the current Aichi Target 15 and the post-2020 biodiversity framework for ecological restoration and recovery of threatened species.

## Materials and methods

Conservation actions in the Red List are categorized based on the hierarchical lexicon developed by the IUCN and the Conservation Measures Partnership [27], where Species Re-introduction is classified as an action under Species Management. According to the Red List, Species Re-introduction includes both conservation translocations that release animals into historical (reintroductions) and non-native (benign introductions) ranges [9]. So, hereafter, to prevent confusion, “re-introduction” will be called “CT” or conservation translocation, and “reintroduction” will only refer to those projects releasing animals to their historical range [16]. Threatened and Near Threatened species on the Red List with CT as a recommended conservation action were identified based on a query of the Red List threat assessment data [9]. These data were analyzed based on CT in general and then separated into reintroductions and benign introductions to investigate any potential differences in habitat quality status for these two groups.

The list of species was filtered to include only amphibians, birds, and mammals that live in terrestrial habitats (241 species). The list of species was further filtered for those that are reliant on forest ecosystems (forest dependent species). Sixty-three percent of the species with CT recommended were forest dependent species. The list was further filtered to exclude species that are not forest dependent based on life history data, such as the lion (*Panthera leo*) and American bison (*Bison bison*) (152 species). The Red List data did not distinguish between subspecies that are forest dependent and those that are not, so those species were removed. Threatened and Near Threatened species were listed as Extinct in the Wild (EW), Critically Endangered (CR), Endangered (EN), Vulnerable (VU), and Near Threatened (NT). Species listed as Data Deficient (DD) or Least Concern (LC) were not included.

Data on the distributions of terrestrial mammals, amphibians, and birds were obtained in November 2019 from BirdLife International’s Handbook of the Birds of the World [28] and the Red List [29]. Data were aggregated at a scale applicable for species management using a global grid of equal area, equal shape hexagons with a 10 km^2^ resolution within 50 degrees of the equator using the R-package ‘dggridR’ [30] in R version 3.6.2 [31]. Areas further than 50 degrees from the equator were dropped from the analysis due to the lack of species with CT recommended at these latitudes. The attributes of presence and origin are associated with each species’ range polygon and were assessed to only include species that were extant and probably extant, attribute value of 1 and 2, respectively, as well as native or reintroduced, attribute value of 1 and 2, respectively. In the case for species considered extinct in the wild, we used ranges classified as extinct, presence attribute of 5. Additionally, portions of ranges that were classified as ‘passage’ or ‘uncertain’ were excluded. The ranges of threatened and Near Threatened species with CT as a recommended conservation action were mapped in QGIS v. 3.10 [32]. Range maps were overlaid with a global terrestrial hexagon layer to count the number of selected species in each 10 km^2^ hexagon. The 10km^2^ resolution highlights areas of high conservation value, which tend to diminish at larger resolutions, especially in isolated patches likely caused by anthropogenic influences such as fragmentation [33].

To calculate the extent of current intact forest and reforestation potential, we used maps from Griscom et al. [34] where they modified a 1 km resolution map from the Atlas of Forest Landscape Restoration Opportunities (FLRO), which takes an estimate of potential forest cover [35, 36], and identifies existing forests [37] and areas that are not compatible with forests restoration, such as deserts, grasslands, dense human populations, cropland, etc. [38, 39]. The average percent of forest cover for each species was calculated using a 1 km resolution aggregated forest cover map [37].

To assess each species’ current and potential forested habitat area, we calculated the percent of a species range that overlaps with areas of current forest extent and restoration potential. We also assessed the amount of a species’ range in a protected area by calculating the percent of a species range that overlaps with a protected area, based on a spatial overlap between polygons for protected areas from the World Database on Protected Areas [40], categories I-VI. Due to the non-normality of the data, a Mann-Whitney U test was used to examine whether the percentages of range within a protected area differed for species recommended for benign introduction and species recommended for reintroduction (*p* < 0.05). This test was performed in R version 3.6.2 [31].

We also assessed the Red List recommended conservation actions of Invasive/Problematic Species Control (hereafter ‘invasive species control’) and Habitat and Natural Processes Restoration (hereafter ‘habitat restoration’) for the species that have CT as a recommended conservation action. To examine the relationship between CT category (benign introduction and reintroduction) and conservation action recommendation (invasive species control and habitat restoration), we used a Chi-square test of independence via R version 3.6.2 [31] (*p* < 0.05). A Chi-square test was deemed suitable after meeting its assumption (80% of the expected values are > 5). Additionally, we assessed whether each species has an active CT program in place, i.e. animals that have been released, and a general recovery plan. For evidence of active species CT, we searched Red List data in addition to examining the first two pages of Google Scholar search engine results using the keywords “reintroduction” and “translocation” plus the species’ name. To assess whether each species has a recovery plan, we used the Red List data to determine if an Action Recovery Plan was in place.

## Results

Ninety bird, 52 mammal, and 12 amphibian threatened and Near Threatened forest dependent species have CT as a recommended conservation action (Table 1; also see S1 Table for a full list of species). Ten species are CR yet have not been seen in the wild since 2009 but have populations in captivity. The last time these species were seen in the wild ranges from 1972 to 2009. Another five species are EW and only remain in captivity. The remaining species are listed as CR, EN, VU, or NT (Table 1).

**Table 1:** Forest dependent threatened and Near Threatened species with CT as a recommended conservation action on the IUCN Red List.

Class	Critically Endangered	Endangered	Extinct in the Wild	Near Threatened	Vulnerable	Total
Amphibia	5	3	-	2	2	12
Aves	29	27	5	4	25	90
Mammalia	8	17	-	8	17	50
Total	42	47	5	15	45	152

Data collected from IUCN Red List on May 22, 2020.

**Fig 1:**
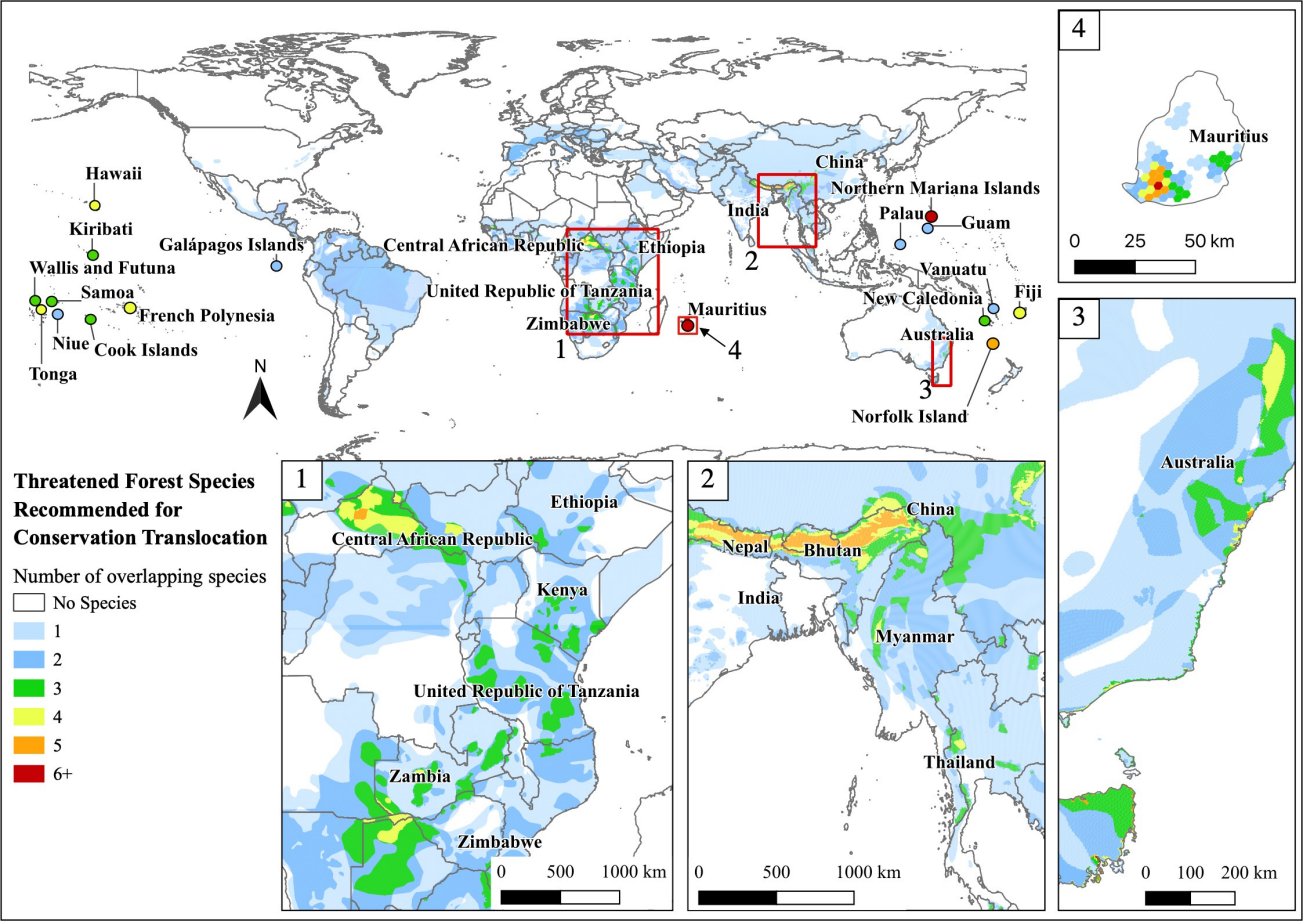
Overlapping species’ range maps for 152 threatened and Near Threatened terrestrial species, amphibians, birds, mammals, that live in forest habitat with CT as a recommended conservation action. Reprinted partially from the Environmental Systems Research Institute (ESRI) under a CC BY license, with permission from ESRI, original copyright 2021.

Eighty-three CT recommended species have restoration potential in their range and overlap with at least one other species (S2 Table). The island of Mauritius, central Africa, eastern Australia, and the Himalayan Mountains region had areas with the highest concentration of threatened, CT recommended species’ overlapping ranges (Fig 1). Fifty-one percent of the CT species live on islands, and the other 49% on the mainland. Eighty-nine percent of species recommended for benign introductions live on islands while only 39% of species recommended for reintroductions live on islands. One hundred and seventy countries have at least one threatened or Near Threatened species with CT recommended. Thirty-nine species have ranges that overlap with multiple countries. Australia has the most species with CT recommended, 25, followed by New Zealand, 16 species, French Polynesia, 14 species, The United States (mostly Hawaiian Islands) and China both have 12 species, and Mexico with 10 species.

Forty-four percent of CT recommended forest species have ranges in a protected area, with existing forest cover, and no invasive species risk. Therefore, 56% of CT recommended forest species currently need habitat management or protection or invasive species control. Twenty-two percent of species have no protected area within their range, 41% are recommended for habitat restoration, and about 47% are recommended for invasive species control. An average of 46% of CT species’ ranges are covered by forest; 44% for reintroductions and 61% for benign introductions. However, we could not calculate forest cover for 25% of species due to the data restrictions where some small oceanic islands were not assessed in the Hansen et al. [37] analysis. Thus, the forest cover estimates could be inflated, especially for benign introductions where nearly 60% of the data, 21 species, for forest cover are missing.

**Fig 2:**
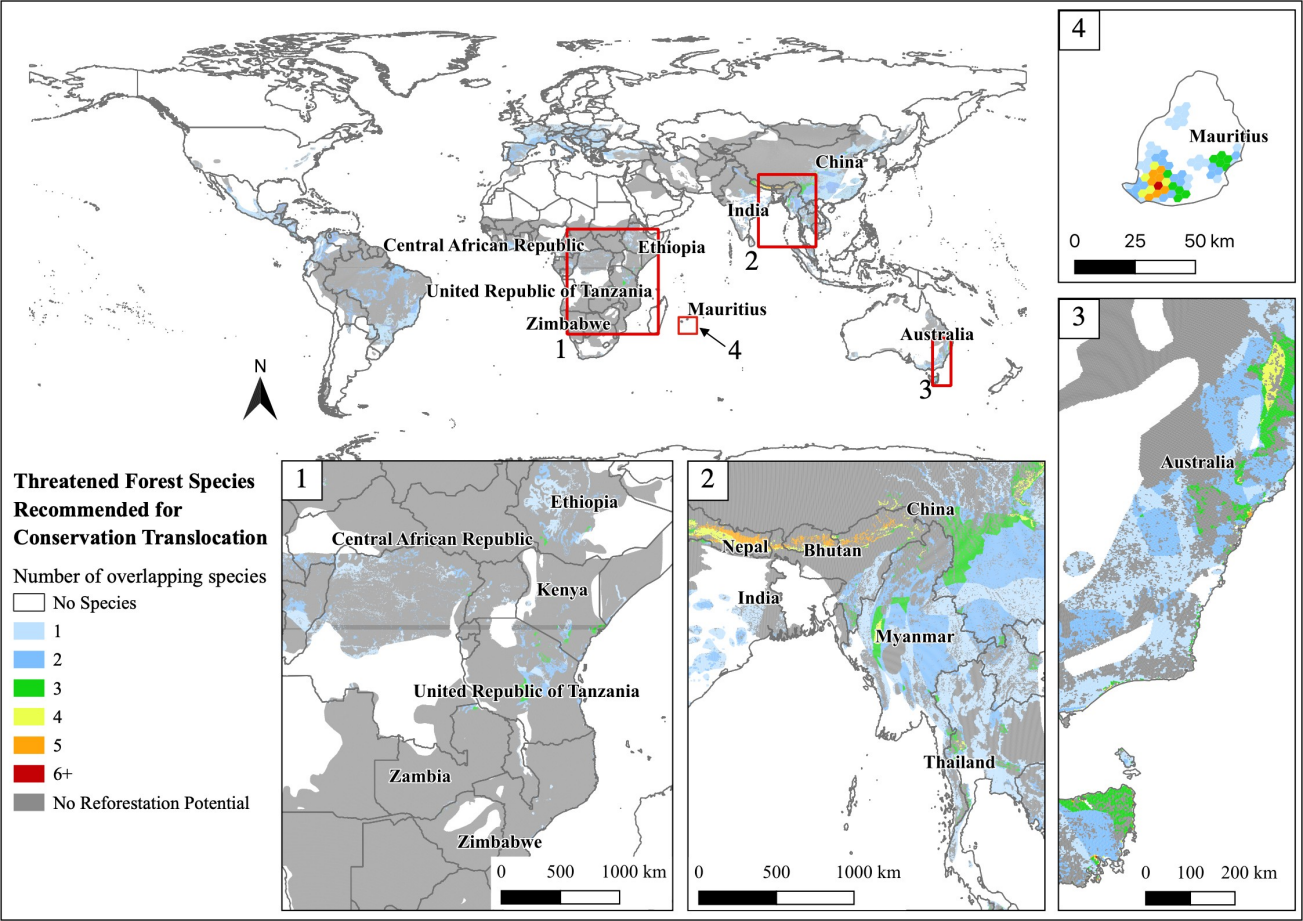
Overlapping species range maps with restoration potential for 152 threatened and Near Threatened terrestrial species, amphibians, birds, mammals, that live in forest habitat with CT as a recommended conservation action. Areas in grey indicate where restoration species’ home ranges exist, but there is no potential for habitat restoration. White areas indicate where no species with recommended CT occur. Reprinted partially from the Environmental Systems Research Institute (ESRI) under a CC BY license, with permission from ESRI, original copyright 2021.

Seven percent of species have greater than 50% of their range with forest restoration potential, and 36% of species have no reforestation potential in their existing range. However, if split, nearly 60% of species recommended for benign introduction have no reforestation potential in their existing historical range, while only 28% of reintroductions have no restoration potential. Mauritius, the Himalayan Mountains, and eastern Australia were the only regions with four or more species with overlapping ranges and forest restoration potential; central Africa only has three or fewer species with overlapping ranges and forest restoration potential (Fig 2). Within these regions, a total of 101,346 km^2^ have four or more species with overlapping ranges and forest restoration potential (Table 2).

**Table 2:** The area within each country with 4 or more species that have overlapping ranges and habitat restoration potential.

Country	Area w/ 4 Species (km^2^)	Area w/ 5 Species (km^2^)	Area w/ 6 Species (km^2^)	Total area per country (km^2^)
Australia	6121	591	64	6775
Bhutan	3541	8172	93	11806
China	14306	84	-	14390
India	5444	9987	67	15498
Myanmar	5131	-	-	5131
Nepal	29967	16912	-	46879
Thailand	868	-	-	868
Total	65375	35747	224	101346

**Fig 3:**
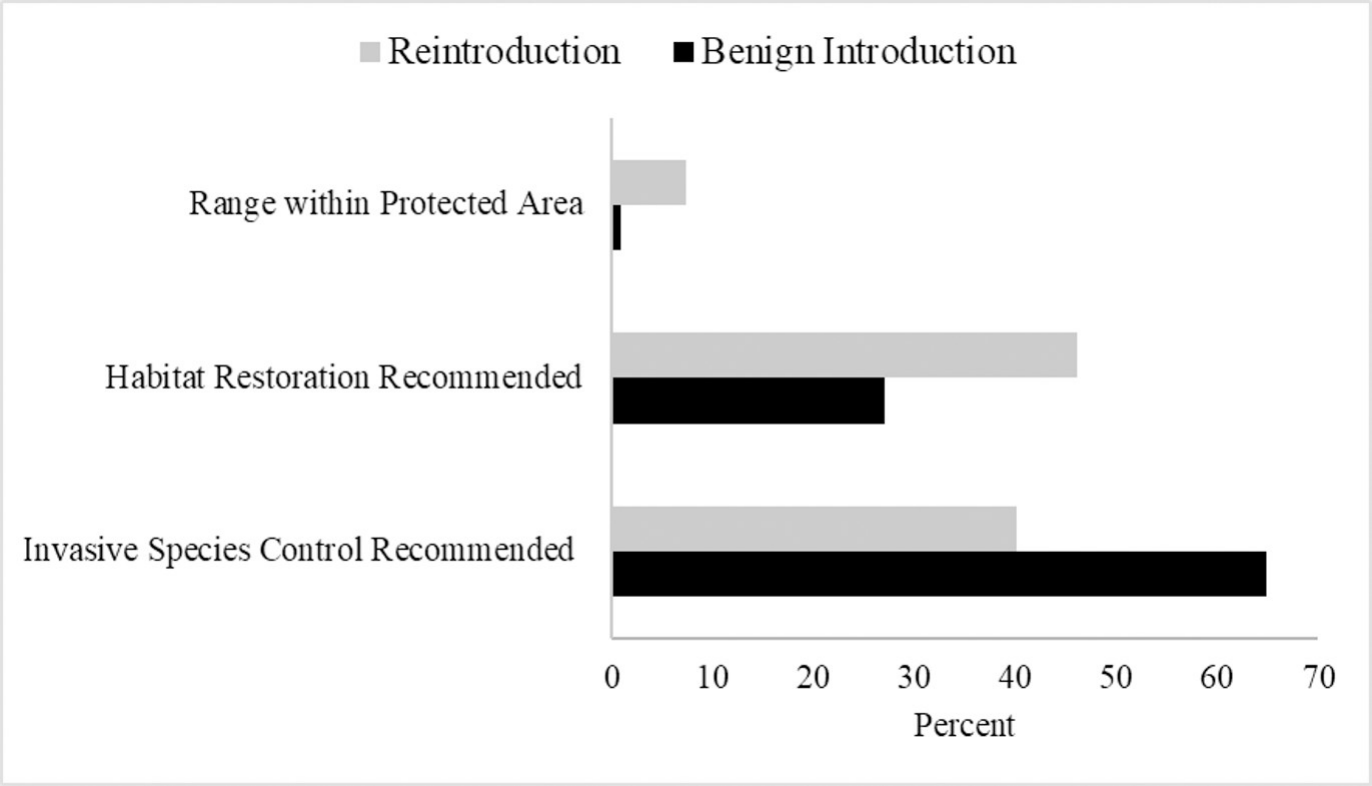
Habitat protection and quality differences for species with reintroduction and benign introduction recommended. Differences in the median percent of species’ ranges within a protected area and the percent of species recommended for habitat restoration and invasive species control. A Mann-Whitney U test revealed the distributions of species range percentage within a protected area was significantly different for reintroductions (n = 115) and benign introductions (n = 37) (*p* = 0.044). A Chi-square test of independence revealed benign introductions were recommended for invasive species control significantly more than expected (χ^2^ = 5.9405, df = 1, *p* = 0.015), but no significant differences between CT category and species recommended for habitat restoration (χ^2^ = 3.4833, df = 1, *p* = 0.062).

Seventy-eight percent of CT recommended species have ranges that are at least partially in protected areas. The distributions of range percentage within a protected area were significantly different for reintroductions and benign introductions (*p* = 0.044), with 7.4% and 0.9% of the ranges protected for species recommended for reintroduction and benign reintroduction, respectively (Fig 3). Twenty-two percent of species do not have ranges that overlap with existing protected areas.

Forty-one percent of the forest dependent species with CT as a recommended conservation action also had habitat restoration as a recommended conservation action with a relatively equal split 46% and 54% on islands and continents, respectively. There was no significant difference in habitat restoration recommendations for species recommended for benign introductions or reintroductions (χ^2^ = 3.4833, df = 1, *p* = 0.062; Fig 3). Invasive species control was recommended for 47% of CT recommended species, 76% of which live on islands. Species recommended for benign introductions had significantly more invasive species control recommended than expected compared to species recommended for reintroductions (χ^2^ = 5.9405, df = 1, *p* = 0.015; Fig 3).

**Fig 4:**
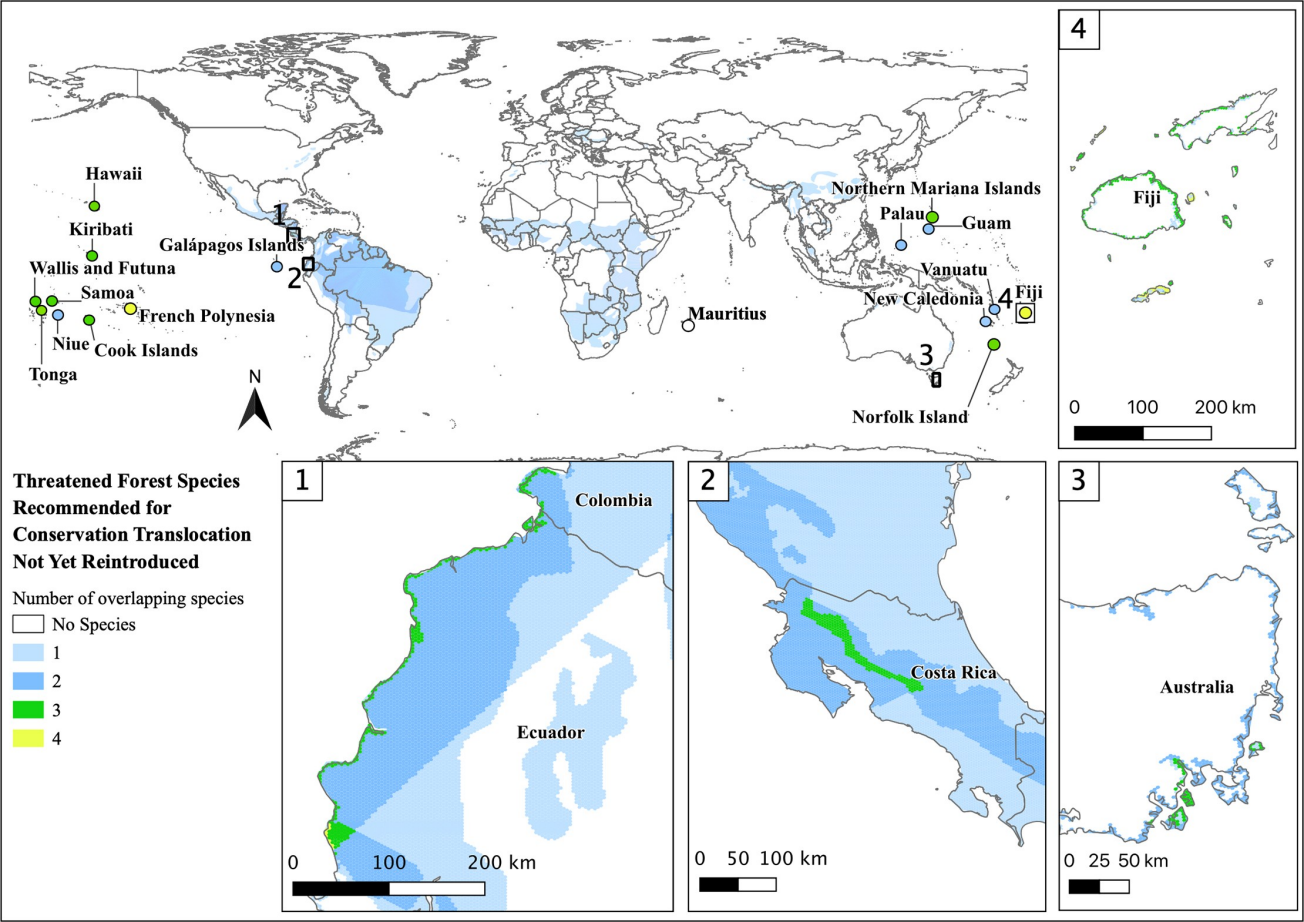
Overlapping species range maps for 68 threatened and Near Threatened terrestrial species, amphibians, birds, mammals, that live in forest habitat without a CT program in-place. Reprinted partially from the Environmental Systems Research Institute (ESRI) under a CC BY license, with permission from ESRI, original copyright 2021.

Exactly half of the 152 species have an Action Recovery Plan in place and 47% of these are mainland species. Forty-five percent of the 152 species do not have an active CT program and of these species, 44% are mainland species (Fig 4). Yet, only 30% of the 152 species have both an Action Recovery Plan and a CT program, which was split equally between mainland and island species. Compared to those without active CT programs, mainland species with CT programs had slightly lower mean percent forest cover (50% versus 32% respectively) and percentage of range with restoration potential (19% versus 16% respectively).

## Discussion

Nearly half of the forest dependent species that have CT recommended have intact forest that is protected and no threat from invasive species in their former ranges indicating that they could be good candidates for reintroduction. For the other 56% of forest dependent species with CT recommended, there is low forest cover, no site-level protected areas, and persistent threat from invasive species in their historical ranges indicating that benign introductions to other locations could be considered. While these results stem from a global analysis and indicate that habitat quality most likely needs improvement for most species recommended for CT, species- and site-specific assessments should always be made before any CT. Our methods take a holistic approach to identify global patterns in the necessity for habitat quality assessments prior to CT actions.

By illustrating areas that would benefit from single or multi-species reintroduction and habitat restoration, we support efforts that incorporate both in conservation project planning as these techniques should be two integral parts of the same goal [41]. Additionally, our data can aid in the habitat modeling process during the development of these restoration and CT plans. More *a priori* objectives can be outlined with the inclusion of our species and landscape data. Detailed statistical correlative or expert-based modeling could be expanded to include the needs of multiple species, climate change, and any nuanced requirement to increase CT success [42].

Central Africa, the Himalaya region, east Australia, and many oceanic islands, especially Mauritius, were identified as important sites for forest restoration, potential land protections, and multi-species reintroduction. These locations coincide with some of the world’s biodiversity hotspots and areas with high endemism [43, 44]. Unfortunately, these are also areas that may continue to see dramatic land-use changes and habitat loss [4, 44–47], thus benefiting from habitat restoration and protection.

Due to the difficulty of execution, current trends in CT science are strongly biased towards single species translocation [41, 48, 49]. Though a leading question in the CT field is what effect the translocated species will have on the ecosystem, and perhaps could the ecosystem benefit from multi-species reintroductions [50], it is rare for a CT program to involve more than one species [51]. Yet, species loss and extinction rarely happen in isolation [52]. Moreover, species that have strong ecological interactions with other species may depend on those species’ existence in the release area [51]. With the concentration of forest restoration potential in some regions of the world, multi-species reintroductions may be possible and larger conservation goals can be met.

Most species’ ranges are contained within a single country (74%). This may reflect the fact that 51% of our 152 species live on islands and many are not migratory. Australia and a few countries in Asia and Africa have the most area suitable for restoration with the potential for multiple species CT. Nepal has the most area for restoration and multi-species CT (Table 2) and has been working for the past few decades in turning forest land back to local communities for management, showing positive changes in forest cover from 1989 to 2001 [53]. Tanzania was also identified as another country with large areas (totaling 20,876 km^2^) available for restoration and multi-species CT for three species (S3 Table). In Tanzania, intelligent restoration design can aid species recovery [54]. With an active community and government participation, around 77% of the matrix forest ecosystem of the Eastern Arc Mountains in Tanzania can be reconnected with only ~8,000 ha of restoration [55]. The addition of these restored forest corridors could also fortify some species’ populations against climate change as restoration in some of Tanzania’s larger fragments would allow upslope movement [54]. The cost and space needed to make considerable improvements in forest structure within these countries, increasing the possibility of multi-species reintroduction, may prove minimal with intelligent design and active participation of governments and local communities.

Of the 152 CT recommended species, about half have CT programs in place or species-specific conservation action plans and only 30% of species have both. Mainland species with CT programs in place, 44 species, had less mean percent forest cover (32%) and restoration potential (16%) than those species without programs in place. It will be important to observe if these CTs prove successful and produce sustainable populations as they might highlight the minimum habitat quality requirements for the translocation of forest dependent species. However, it is not known whether 32% forest cover could sustain these initial translocated populations or be adequate to grow the population if needed. For example, reintroduction of the red-billed curassow (*Crax blumenbachii*) illustrated that though forest cover is essential to their persistence, they could also endure in the presence of other less suitable habitats, such as pastureland, which provides hope for their survival in the modern mosaic of modified habitats [56]. Regardless, 32% forest cover may be useful for CT programs initially, but habitat restoration should be implemented in tandem with these efforts if increasing populations back to their original extent is the goal.

**Fig 5:**
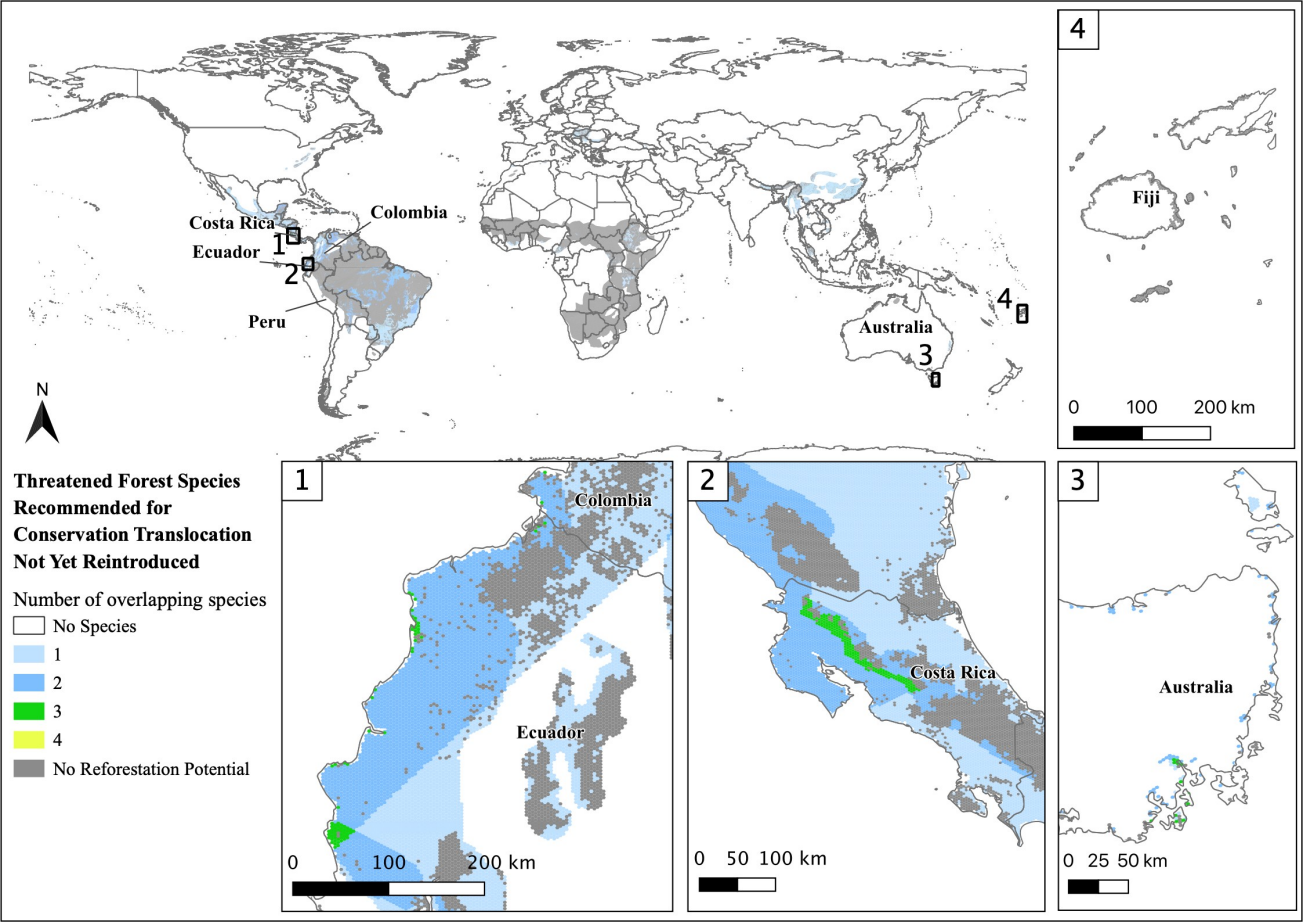
Overlapping species range maps with restoration potential for 68 threatened and Near Threatened terrestrial species, amphibians, birds, mammals, that live in forest habitat without a CT program in-place. Areas in grey indicate where restoration species’ home ranges exist, but there is no potential for habitat restoration. White areas indicate where no species with recommended CT occur. Reprinted partially from the Environmental Systems Research Institute (ESRI) under a CC BY license, with permission from ESRI, original copyright 2021.

The mainland species without current CT programs have an average of 50% of their range with forest cover, arguably making them good candidates for CT. Also, these species have ranges with an average restoration potential of 20%. If habitat restoration projects are successful and done strategically, up to 70% of these species’ ranges would have forest cover with the potential to sustain large populations. The highest concentration of these species is in Central and South America (Figs 4 and 5) which is likely due to the high endemism and extensive habitat loss [43–47]. For example, the Alagoas antwren (*Myrmotherula snowi*) lives in the highly fragmented northeastern portion Atlantic Rainforest in Brazil and could benefit greatly from both CT and forest restoration [57]. This species was shown to have a high percentage of range restoration potential (63%; see S1 Table) suggesting habitat restoration a main priority before CT program implementation. Restoring the connections between forest fragments to make larger patches of forest could require minimal habitat restoration effort, increasing contiguous forest significantly [54]. Yet, this would be a large undertaking for one species. Though maximal conservation efforts should be implemented for each species, if conservation resources are low, starting with areas that could benefit from multi-species CT could increase access to funding and other necessities. Ecuador and Costa Rica have areas with habitat restoration potential for multiple species without CT programs in place (Fig 5). In Costa Rica, the oncilla (*Leopardus tigrinus*), margay (*Leopardus wiedii*), and yellow-naped amazon (*Amazona auropalliata*) have overlapping ranges with habitat restoration potential. As charismatic species, combining these three species’ conservation resources may increase the likelihood for restoring habitat, creating protected areas, and starting CT programs for eventual release.

Releasing animals into a protected area is another piece of the complicated puzzle leading to CT success. Our results show that just over three-quarters of the 152 species have ranges partially within a protected area. However, only a median of 5.5% and an average of 13% of species’ range recommended for CT was within a protected area, benign introductions only having a median of 0.9% of their range protected, and reintroductions with a median of 7.4%. Though low, these range percentages within a protected area are not surprising when the median size of protected areas globally is only 0.45 km^2^ [40].

Having the release site and much of the release area within a protected location likely has real implications for the species’ establishment and long-term persistence. The Eurasian lynx (*Lynx lynx*) reintroduction program saw an increase in lynx density in areas closer to national parks due to increased forest cover and prey density and no threat of illegal killing [58]. Though increasing the amount of protected area within a species’ range is difficult due to many political or socioeconomic factors, the land we suggest for restoration could provide more opportunities to increase protected spaces. For those species prone to frequent and wide roaming within their range, protected areas act as reservoir population sources surrounded by sinks due to anthropogenic threats [59]. It is not ideal to release animals without their threats fully dealt with; however, the complete eradication of all threats is often unlikely. If the only other option is to hold the species in captivity, which is expensive and can lead to animals with maladapted behaviors [20, 60], releasing them into a protected area is a viable option. Therefore, increasing the amount of protected area within a species range to above the low values seen here, potentially with the land we are suggesting for restoration, could increase the likelihood of population persistence. Yet, even if extensive forest restoration and protection projects are successfully implemented, other restoration needs, such as invasive species risk, within the ecosystem may need attention before reintroductions take place. If the ecosystem cannot be restored to an appropriate level for the species, then benign introduction could be an option.

Our results bolster the argument that invasive species risk, especially for island species, is a leading factor pushing species to extinction [61–63]. For example, the Mauritius fody (*Foudia rubra*) was introduced to a novel, predator-free island in the early 2000s with some successful population establishment and persistence [64, 65]. Even though our results show that Mauritius has the potential for forest restoration (Fig 2), much of its conservation depends on the continued control of invasive mammalian predators [64]. Like Mauritius, most threatened island species have invasive species inhabiting their island [66]. Due to the technical and financial complications of eradication, continued conservation intervention is likely, and these species may ultimately be conservation dependent [66]. Many islands have the potential for multi-species conservation because of the high concentration of species recommended for CT without current CT programs in place (Fig 4). However, most of these species are recommended for benign introductions due to the invasive species risk and low habitat restoration potential. Islands may have the most potential for CT implementation, but face the most risk and barriers to successful conservation action.

Even without current invasive species control recommendations, many species need or will need a form of management that is related to invasive species control. Islands without invasive species will need to maintain strict biosecurity measures, which are also complicated and costly [66]. For example, invasive species control is not currently recommended for the endangered hihi or stitchbird (*Notiomystis cincta*), however, that is only because they exist on predator-free islands and their populations rely on continued management [67]. Due to the difficulty of invasive species control, more conservation planners might lean toward benign introductions for species endemic to islands. It might be more feasible to be watchful and prevent invasive species from entering an island than to eradicate them once they are already in place.

Benign introductions have received increased attention in the past two decades for both their potential benefits but also risks involving wildlife conservation [68]. Benefits include preserving species on the brink of extinction from pervasive challenges such as human encroachment and climate change. However, there is a risk of introducing a potentially invasive species [50, 69]; consequently, extensive research is needed on the use of benign introductions. Here, benign introductions are recommended primarily for avian, island species living in areas with little-to-no restoration potential, minimal protection (median 0.9%, mean 12% range within protected areas), and in need of invasive species control (Fig 3; also see S1 Table). However, our forest cover layers may not provide the necessary information for many of the species on small oceanic islands as our data did not include forest cover information for small range islands (see Hansen et al. [37]). Future research should specifically investigate forest cover on smaller islands, restoration needs, and ultimately the likelihood of maintaining the island for the long-term persistence of its wildlife. Lastly, our results do not conclusively point to the need for benign introductions, but for islands with compounding anthropogenic threats, they may become a more popular crisis conservation solution.

A large hurdle in the CT planning process is the lack of evidence-based information and biases within the field [15]. Our results are aligned with the taxonomic bias frequently seen in conservation biology [70]; avian species make up around 58% of our group, with mammals at 32% and amphibians making up the rest (Table 1). Passerines dominate the list, while artiodactylids, parrots, pigeons and doves, marsupials, and carnivores are the second most common. In reintroduction science, mammal related projects, particularly artiodactylids and carnivores, are over-represented [71]. The overrepresentation of charismatic species, such as the giant panda (*Ailuropoda melanoleuca*), the Persian fallow deer (*Dama mesopotamica*), and the orange-bellied parrot (*Cyanoramphus malherbi*), are common in both conservation biology and reintroduction science [70, 71]. This is not to say the work done to protect these species is not important; these species’ conservation may serve as a type of umbrella, passively providing protections for the smaller, less charismatic species in the same region [72]. However, the umbrella species concept is contested [73]. Our results point to areas of not just one, but multiple species’ ranges that could provide a much larger umbrella, suggesting the need for more regional/ecosystem-based conservation.

The taxonomic bias issue may also be due to reporting errors. The Red List is a comprehensive list of species conservation information; however, reintroduction or general conservation information, especially for the lesser-known species, is likely missing. Additionally, some endangered species projects may not suggest CT as a viable option due to time, spatial, and/or monetary constraints, which are very common in this field, especially if captive breeding is needed [74–76]. With the impending effects of climate change and the current habitat degradation, the need for complete, up-to-date species conservation information is critical. Therefore, our list is likely missing critical information and should not be considered conclusive, but instead the beginning of selecting areas for habitat restoration and large-scale CT.

## Conclusions

CT techniques are risky, yet the complex nature of threatened species conservation demands complex, novel solutions, and assessing multiple aspects of habitat quality, such as available forest, invasive species concerns, and protection, in advance of CT efforts should bolster the success of such efforts. Any translocation should not only consider the ramifications of each release but how these methods affect the species and ecosystem [77]. We suggest several regions where multiple species could be reintroduced together to restore the functional roles within their ecosystem. The potential use of these areas for forest restoration, protected area expansion, eradication of invasive species, and the reintroduction of multiple species could further the application of landscape-level research and conservation.

## Supporting information

S1 Table Fully compiled raw species and habitat data table. (XLSX)

S2 Table List of species with range overlap and habitat restoration potential. Range overlap with one or more separate species.
(XLSX)

S3 Table List of species with range overlap and habitat restoration potential. Range overlap with three or more separate species.
(XLSX)
